# Analysis of Tryptophan Metabolic Profile Characteristics and Clinical Value in Differentiated Thyroid Cancer Patients

**DOI:** 10.1002/cam4.70808

**Published:** 2025-03-25

**Authors:** Qiang Miao, Xinhua Dai, Xiaojuan Wu, Li Luo, Junlong Zhang, Han Luo, Bei Cai

**Affiliations:** ^1^ Department of Laboratory Medicine West China Hospital, Sichuan University Chengdu Sichuan China; ^2^ Sichuan Clinical Research Center for Laboratory Medicine Chengdu Sichuan China; ^3^ Clinical Laboratory Medicine Research Center of West China Hospital Chengdu Sichuan China; ^4^ Division of Thyroid and Parathyroid Surgery West China Hospital, Sichuan University Chengdu Sichuan China

**Keywords:** biomarkers, differentiated thyroid cancer, LC–MS/MS, metabolomics, tryptophan metabolism

## Abstract

**Background:**

Differentiated thyroid cancer (DTC) is the primary subtype of thyroid cancer. Timely diagnosis and intervention are crucial for improving prognosis and survival. However, the effectiveness of existing serum markers is limited, necessitating the discovery of new biomarkers.

**Methods:**

This study utilized liquid chromatography–tandem mass spectrometry to analyze tryptophan metabolic profiles in serum samples from 105 DTC patients and 50 healthy controls. Independent predictors of DTC were identified through univariate intergroup comparisons and multivariate logistic regression analysis, leading to the development and validation of a new diagnostic model.

**Results:**

Significant differences were observed in 11 tryptophan metabolites between DTC patients and controls. Logistic regression identified nicotinamide, 3‐hydroxyanthranilic acid, 5‐hydroxytryptophan, melatonin, and indole‐3‐propionic acid as independent predictors. The nomogram prediction model was established based on these five metabolites, and according to the Hosmer–Lemeshow test, the model showed good fit. The five‐metabolite diagnostic model demonstrated 84.8% sensitivity, 90.0% specificity, and an area under the ROC curve of 0.932. Decision curve analysis indicated that the model had significant advantages over serum thyroglobulin.

**Discussion:**

Tryptophan metabolism exhibits distinct changes in DTC patients, with specific metabolites serving as early diagnostic markers. The five‐metabolite panel demonstrates potential for improving early detection and management of DTC.

## 1 Introduction

Thyroid carcinoma (TC), a malignancy originating from the thyroid follicular epithelium or parafollicular C cells, is the most common neoplastic lesion in the head and neck region [1, 2]. The global incidence of TC has risen significantly in recent years, making it the ninth most prevalent malignant tumor worldwide [3, 4]. According to the latest report from the National Cancer Center in 2022, the number of new TC cases in China has surged to 466,100, ranking third among all malignant tumors and underscoring its profound impact on public health in China [5]. TC is histologically classified into three main types: differentiated thyroid cancer (DTC), medullary thyroid cancer, and undifferentiated thyroid cancer. DTC, which includes papillary, follicular, and Hürthle cell carcinomas, is the most common subtype [6, 7, 8]. Although DTC generally has a favorable prognosis, delayed diagnosis can lead to disease progression and reduced survival rates, particularly in patients who are resistant to radioactive iodine therapy and at a higher risk for recurrence and distant metastasis [9, 10]. The 5‐year survival rate for TC in China is 84.3%, which is lower than that in European and American countries [11]. This disparity is primarily attributed to the lack of early‐stage cases and the low rate of early detection. Therefore, timely diagnosis and intervention are crucial for improving patient outcomes and increasing survival statistics [12].

Individuals with TC often remain asymptomatic. A retrospective multicenter study found that only 30% of patients exhibit symptoms at diagnosis and those who do typically present with neck masses, dysphagia, a sensation of a foreign body, or hoarseness, which are associated with more advanced disease stages [13]. The current serum markers for the identification of DTC are thyroglobulin (Tg) and thyroid‐stimulating hormone (TSH); however, these markers have inherent limitations in the early diagnosis and ongoing monitoring of the disease [14, 15]. As a result, the discovery of novel biomarkers is crucial for enhancing early detection and therapeutic intervention in TC. Metabolomics, an emergent field within the “omics” sciences, provides new insights into disease diagnosis and treatment by analyzing the small molecule metabolites present in biological specimens [16]. Tryptophan, an essential amino acid, is integral to a wide range of physiological processes, including immune modulation, cellular proliferation, inflammatory responses, metabolic pathways, and neurological functions, primarily via the kynurenine pathway and serotonin synthesis [17]. Evidence suggests that tryptophan and its metabolites significantly influence the tumor immune microenvironment and facilitate tumorigenesis [18, 19]. Notably, alterations in tryptophan and its metabolites have been detected in thyroid cancer patients, suggesting their potential as distinctive metabolic signatures of the disease [20, 21]. Exploring tryptophan metabolism profiles introduces innovative avenues for the early diagnosis of TC, with the variable expression of these metabolites offering the potential for a noninvasive and cost‐effective diagnostic strategy. This approach could enhance existing diagnostic methodologies and contribute to the refinement of clinical management protocols. Despite previous research documenting alterations in tryptophan metabolite expression patterns within thyroid tumors, a systematic and comprehensive investigation into the tryptophan metabolic profile specific to DTC is still lacking [22, 23]. This study aims to address this gap by conducting a targeted metabolomic analysis of the tryptophan metabolic profile in serum samples from DTC patients. The objective is to identify novel biomarkers and develop predictive models that could provide more accurate indicators for the noninvasive early diagnosis and therapeutic monitoring of DTC.

## 2 Method

### 2.1 Study Subjects

The subjects included in this study were mainly thyroid cancer patients who underwent surgical intervention in the Department of Thyroid Surgery of our hospital from June 2022 to December 2023 and healthy subjects who underwent physical examinations during the same period. Inclusion criteria included individuals aged 18 years and above with a histopathologically confirmed diagnosis of DTC undergoing first‐time surgical intervention. Exclusion criteria included individuals under 18 years (due to differences in metabolic profiles between children and adults); those with severe hepatic or renal diseases (as these conditions significantly alter the metabolism and clearance of tryptophan and its derivatives); those with coexisting malignant neoplasms (to ensure metabolic changes were specifically related to DTC); those with a recent history of fractures or surgeries (as inflammation and metabolic changes induced by trauma could affect study results); women in the gestational or lactational phase (due to hormonal changes that could influence metabolism); and individuals adhering to specific dietary regimes such as vegetarianism or glutamate‐free diets (as diet significantly impacts tryptophan intake and metabolism). Additionally, participants with gastrointestinal symptoms were excluded to avoid potential metabolic alterations affecting tryptophan metabolism, as well as those with a history of obesity, hypertension, or dyslipidemia, as these conditions could influence metabolic profiling. Finally, individuals who had taken antibiotics or probiotics within the preceding 2 months were excluded to prevent confounding effects on gut microbiota and tryptophan metabolism. This study received approval from the Ethics Committee of West China Hospital, Sichuan University, and adhered to the Helsinki Declaration; all participants provided informed consent.

### 2.2 Research Methods

The remaining serum after routine clinical testing was collected and stored in 1.5‐mL Eppendorf tubes at −80°C. Clinical data and relevant test results, including patient age, gender, serum Tg, thyroid ultrasound findings, and postoperative pathology examination results, were concurrently gathered. Clinical stage (cTNM) and pathological stage (pTNM) were established based on preoperative assessment and postoperative pathology findings, utilizing the American Joint Committee on Cancer (AJCC) staging system (8th edition) [24]. Liquid chromatography–tandem mass spectrometry (LC–MS/MS) was employed to detect 19 tryptophan metabolites in patient serum. A comparison was made of the differences in tryptophan metabolites between patients with DTC and a healthy control group. Based on these differential metabolites, a nomogram for early DTC diagnosis was constructed.

### 2.3 Tryptophan Metabolite Detection

#### 2.3.1 Instruments and Reagents

Serum Tg was detected using electrochemiluminescence immunoassay (Roche cobas e801, Switzerland). The YS EXACT 9900 MD high‐performance liquid chromatography–tandem mass spectrometer (Shandong YingSheng Biotechnology Co. Ltd.) was utilized for the targeted detection of tryptophan metabolites. The Acquity HSS T3 column (2.1 × 100 mm, 1.8 μm, Waters, USA) was employed for chromatographic separation. Other instruments included the BT125D electronic analytical balance (Sartorius, Germany), Vortex‐Genie2 vortex shaker (Scientific Industries, USA), Centrifuge 5418R high‐speed refrigerated centrifuge (Eppendorf, Germany), and DC‐24‐RT nitrogen evaporator (Shanghai Anpu Scientific Technology Co. Ltd.). Detection reagents, including acetonitrile, methanol, dimethyl sulfoxide, and formic acid, were all purchased from Sigma‐Aldrich. Standard reference materials for the 19 tryptophan metabolites and their isotopic internal standards were obtained from Sigma‐Aldrich and Toronto Research Chemicals.

#### 2.3.2 Serum Sample Pretreatment

A volume of 100 μL of serum sample was taken and combined with 20 μL of internal standard working solution and 100 μL of 0.1% aqueous formic acid. The mixture was thoroughly mixed, followed by the addition of 400 μL of acetonitrile, and vortexed for 5 min. Centrifugation was performed at 14,000*g* for 10 min at 4°C. The supernatant (500 μL) was collected postcentrifugation and dried under a nitrogen gas stream. The dried pellet was reconstituted with 100 μL of 0.1% aqueous formic acid, mixed well, and recentrifuged at 14,000*g* for 5 min at 4°C. Finally, 80 μL of the supernatant was prepared for injection and analysis.

#### 2.3.3 Chromatographic and Mass Spectrometry Conditions

Chromatographic conditions: Mobile phase A consisted of a 0.1% aqueous solution of formic acid, while mobile phase B was a 0.1% formic acid in acetonitrile. The flow rate was set at 250 μL/min with an injection volume of 5 μL. A gradient elution method was applied, starting with 98% mobile phase A for the first 8 min, followed by a gradient to 50% mobile phase A from 8 to 9 min, a further gradient to 20% mobile phase A from 9 to 9.2 min, and concluding with a gradient to 2% mobile phase A sustained until 13 min. Mass spectrometry conditions: An electrospray ion source was utilized in positive ion scan mode. The spray voltage was set at 3000 V, with sheath gas (nitrogen) at 45 arb, auxiliary gas (nitrogen) at 16 arb, the ion transfer tube temperature at 225°C, the vaporization temperature at 450°C, and the collision gas (argon) at 1.5 mTorr. Detection was achieved using multiple reaction monitoring modes.

### 2.4 Statistical Analysis

Data analysis was conducted utilizing SPSS 23.0 (IBM Inc., Armonk, NY, USA) and R software (Version 4.2.3). The normality of continuous variables was assessed using the Shapiro–Wilk test and the Kolmogorov–Smirnov test. For continuous data exhibiting normal distribution, the mean ± standard deviation was calculated, with t‐tests employed for between‐group comparisons. Nonnormally distributed continuous data were presented as median (interquartile range), and the Wilcoxon rank‐sum test was applied for between‐group comparisons. Categorical data were expressed as percentages (%) and analyzed using the chi‐square test. Binary logistic regression analysis was conducted to identify an independent tryptophan metabolite for diagnosing DTC patients. In R software, packages including rms, pROC, and decision curve were utilized to construct a predictive nomogram model for early DTC diagnosis. The model's stability was ascertained through 1000 bootstrap resampling repetitions for the nomogram. The model's calibration ability was evaluated using the Hosmer–Lemeshow goodness‐of‐fit test. The receiver operating characteristic (ROC) curves and decision curves were plotted to validate and assess the diagnostic performance of the model. A significance level of *p* < 0.05 was considered to indicate statistically significant differences.

## 3 Results

### 3.1 Clinical Characteristics of the Study Cohort

A total of 155 participants were included in the study, comprising 105 patients diagnosed with DTC and 50 healthy controls. Among the DTC patients, 26 were male and 79 were female, with an average age of 41.3 ± 11.2 years. The control group consisted of 12 males and 38 females, with an average age of 41.2 ± 10.8 years. The fundamental clinical attributes are detailed in Table 1. No statistically significant differences were observed in age and sex between patients with DTC and the healthy control group (*p* = 0.989 and *p* = 0.918, respectively), whereas a significant difference in serum Tg levels was noted (*p* = 0.009). The gender distribution among DTC patients showed a ratio of approximately 1:3 in favor of females. Unilateral nodules were present in 86 DTC patients, and 45 patients had nodules with a diameter exceeding 1 cm. The predominant histological subtype was papillary thyroid carcinoma (PTC), accounting for 77.2% of cases. Patients in stages T1 to T2 constituted 66.7% (70 cases), with 47.6% (50 patients) at N0 stage and 52.4% (55 patients) at N1 stage. Notably, no DTC patient exhibited distant metastasis, as indicated by an M1 stage of 0. A substantial majority, 92.4% (97 cases), of DTC patients were classified at clinical stage I, according to the TNM staging system.

**TABLE 1:** Patient general characteristics.

Features	DTC, (*n* = 105)^a^	HC, (*n* = 50)^a^	*p*
Age, (years)	40 (33, 49)	41 (33, 49)	0.989
Sex			0.918
Male	26 (24.8%)	12 (24.0%)	
Female	79 (75.2%)	38 (76.0%)	
Thyroglobulin (μg/L)	13.6 (7.22, 27.9)	8.71 (5.86, 13.8)	0.009
Tumor location			
Unilateral nodule	86 (81.9%)		
Bilateral nodule	19 (18.1%)		
Tumor diameter (cm)			
≥ 1	45 (42.9%)		
< 1	60 (57.1%)		
Pathological type			
PTC	81 (77.2%)		
FTC	14 (13.3%)		
Mixed (PTC and FTC)	10 (9.5%)		
Clinical T stage			
T1~T2	70 (66.7%)		
T3~T4	35 (33.3%)		
Clinical N stage			
N0	50 (47.6%)		
N1	55 (52.4%)		
Clinical M stage			
M0	105 (100%)		
M1	0		
TNM stage			
I	97 (92.4%)		
II~III	8 (7.6%)		

Abbreviations: DTC, differentiated thyroid carcinoma; FTC, follicular thyroid carcinoma; HC, healthy controls; PTC, papillary thyroid carcinoma.

^a^ Median (IQR) or frequency (%).

### 3.2 Comparative Analysis of Tryptophan Metabolite Levels

Utilizing targeted LC–MS/MS, the serum concentrations of 19 tryptophan metabolites among the study participants were precisely measured. Univariate statistical analysis was applied, and the findings are detailed in Table 2. Comparative analysis against the healthy controls revealed elevated serum levels of quinolinic acid (QA), 3‐hydroxyanthranilic acid (3‐HAA), 3‐hydroxykynurenine (3‐HK), kynurenine (Kyn), tryptophan (Trp), 5‐hydroxytryptophan (5‐HTP), melatonin, and indole‐3‐aldehyde (IAld) in DTC patients. In contrast, decreased levels of nicotinamide, N‐acetylserotonin (NAS), and indole‐3‐propionic acid (IPA), with all differences being statistically significant (*p* < 0.05 for all). Conversely, no statistically significant variations were detected in the serum concentrations of picolinic acid (PA), xanthurenic acid (XA), kynurenic acid (KA), 5‐hydroxytryptamine (5‐HT), 5‐hydroxyindoleacetic acid (5‐HIAA), indole‐3‐lactic acid (ILA), indole‐3‐acetic acid (IAA), and neopterin when comparing the two cohorts (*p* > 0.05 for all). Additionally, no significant disparity was identified in the Kyn/Trp ratio (KTR) between groups (*p* = 0.280).

**TABLE 2:** Comparative analysis of tryptophan metabolite concentrations.

Features	Overall, (*n* = 155)^a^	DTC, (*n* = 105)^a^	HC, (*n* = 50)^a^	*p*
Nicotinamide (nmol/L)	297.28 (231.59, 380.72)	263.65 (211.72, 344.83)	357.08 (306.18, 451.55)	**< 0.001**
QA (nmol/L)	239.02 (192.71, 286.79)	250.44 (204.52, 318.04)	221.97 (178.81, 272.91)	**0.011**
PA (nmol/L)	22.62 (16.49, 29.72)	22.62 (16.4, 30.87)	22.9 (16.65, 28.23)	0.919
3‐HAA (nmol/L)	20.09 (14.86, 27.77)	23.41 (16.19, 31.57)	16.64 (12.47, 19.88)	**< 0.001**
3‐HK (nmol/L)	29.75 (24.05, 35.71)	31.76 (25.69, 40.30)	24.86 (20.13, 30.15)	**< 0.001**
XA (nmol/L)	10.88 (7.31, 14.79)	10.99 (7.14, 15.35)	10.47 (8.11, 14.21)	0.838
Kyn (nmol/L)	1905.38 (1602.04, 2219.53)	1984.97 (1657.71, 2395.77)	1686.81 (1399.16, 2044.19)	**< 0.001**
KA (nmol/L)	42.66 (32.43, 55.38)	42.6 (32.37, 54.86)	43.46 (33.22, 56.54)	0.874
Trp(μmol/L)	57.4 (50.74, 64.48)	59.97 (54.76, 68.41)	50.74 (44.84, 57.13)	**< 0.001**
KTR (nmol/L)	33.34 (28.25, 38.52)	32.92 (27.96, 38.37)	35.34 (28.38, 38.67)	0.280
5‐HTP (nmol/L)	2.89 (2.44, 3.58)	3.32 (2.79, 3.74)	2.35 (1.98, 2.71)	**< 0.001**
5‐HT (nmol/L)	6.52 (3.90, 12.15)	6.16 (3.90, 13.04)	7.16 (4.08, 11.97)	0.826
NAS (nmol/L)	0.039 (0.027, 0.057)	0.034 (0.023, 0.050)	0.055 (0.039, 0.080)	**< 0.001**
Melatonin (nmol/L)	0.06 (0.027, 0.103)	0.079 (0.036, 0.130)	0.027 (0.020, 0.044)	**< 0.001**
5‐HIAA (nmol/L)	27.18 (22.77, 33.71)	27.95 (23.2, 35.31)	25.38 (21.7, 30.51)	0.068
IPA (nmol/L)	768.92 (397.64, 1295.96)	691.72 (330.7, 1084.20)	1131.45 (554.04, 1678.65)	**0.002**
ILA (nmol/L)	594.20 (500.59, 727.24)	595.42 (497.41, 707.66)	580.39 (502.88, 819.03)	0.516
IAA (nmol/L)	1146.93 (901.80, 1415.46)	1175.88 (912.44, 1404.25)	1130.68 (855.50, 1419.13)	0.596
IAld (nmol/L)	163.05 (121.42, 208.80)	184.89 (147.20, 231.15)	109.97 (82.89, 160.72)	**< 0.001**
Neopterin (nmol/L)	4.12 (3.56, 4.76)	4.11 (3.65, 4.77)	4.13 (3.32, 4.73)	0.469

*Note:* Bold indicates statistically significant differences.

Abbreviations: 3‐HAA, 3‐hydroxyanthranilic acid; 3‐HK, 3‐hydroxykynurenine; 5‐HIAA, 5‐hydroxyindoleacetic acid; 5‐HT, 5‐hydroxytryptamine; 5‐HTP, 5‐hydroxytryptophan; DTC, differentiated thyroid carcinoma; HC, healthy controls; IAA, indole‐3‐acetic acid; IAld, indole‐3‐aldehyde; ILA, indole‐3‐lactic acid; IPA, indole‐3‐propionic acid; KA, kynurenic acid; KTR, the Kyn/Trp ratio; Kyn, kynurenine; NAS, N‐acetylserotonin; PA, picolinic acid; QA, quinolinic acid; Trp, tryptophan; XA, xanthurenic acid.

^a^ Median (IQR).

### 3.3 Logistic Regression Analysis of DTC Patients

A logistic regression analysis was conducted incorporating the tryptophan metabolites that demonstrated statistically significant variances between the cohorts. The findings suggest that nicotinamide (OR = 0.987, 95% CI 0.981–0.994), 3‐HAA (OR = 1.105, 95% CI 1.007–1.213), 5‐HTP (OR = 3.812, 95% CI 1.509–9.633), melatonin (OR = 1.109, 95% CI 1.006–1.032), and IPA (OR = 1.002, 95% CI 1.001–1.003) are independent predictors of DTC development, with all associations reaching statistical significance (*p* < 0.05 for all, Table 3).

**TABLE 3:** Multivariate logistic regression analyses related to DTC.

Features	Estimate	Standard error	Odds ratio	95% CI	*p*
Nicotinamide	−0.013	0.003	0.987	(0.981–0.994)	< 0.001
QA	0.007	0.005	1.007	(0.997–1.017)	0.152
3‐HAA	0.100	0.047	1.105	(1.007–1.213)	0.035
3‐HK	−0.023	0.028	0.978	(0.926–1.032)	0.417
Kyn	0.001	0.001	1.001	(0.999–1.002)	0.532
Trp	0.033	0.023	1.034	(0.989–1.081)	0.145
5‐HTP	1.338	0.473	3.812	(1.509–9.633)	0.005
NAS	0.002	0.005	1.002	(0.992–1.011)	0.737
Melatonin	0.019	0.007	1.019	(1.006–1.032)	0.005
IPA	0.001	0.001	1.002	(1.001–1.003)	0.040
IAld	0.001	0.005	1.001	(0.991–1.011)	0.838
Constant	−6.562	2.148	/	/	/

Abbreviations: 3‐HAA, 3‐Hydroxyanthranilic acid; 3‐HK, 3‐hydroxykynurenine; 5‐HTP, 5‐hydroxytryptophan; CI, confidence interval; DTC, differentiated thyroid carcinoma; IAld, indole‐3‐aldehyde; IPA, indole‐3‐propionic acid; Kyn, kynurenine; NAS, N‐acetylserotonin; QA, quinolinic acid; Trp, tryptophan.

### 3.4 Development and Validation of the DTC Diagnostic Model

**FIGURE 1:**
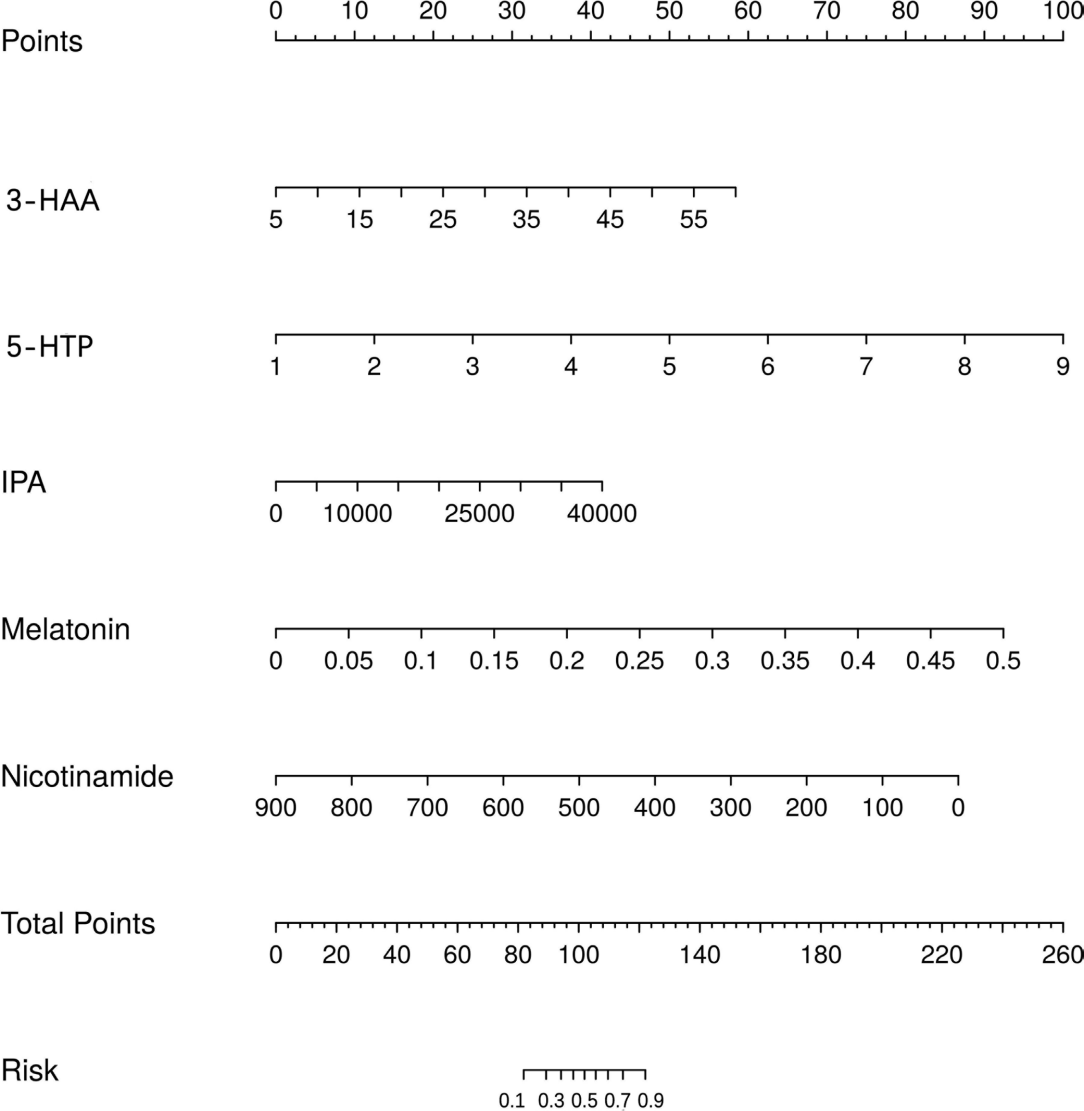
Nomogram for predicting differentiated thyroid carcinoma (DTC). Patients were scored through each item, and total points were calculated to obtain the probability of DTC.

**FIGURE 2:**
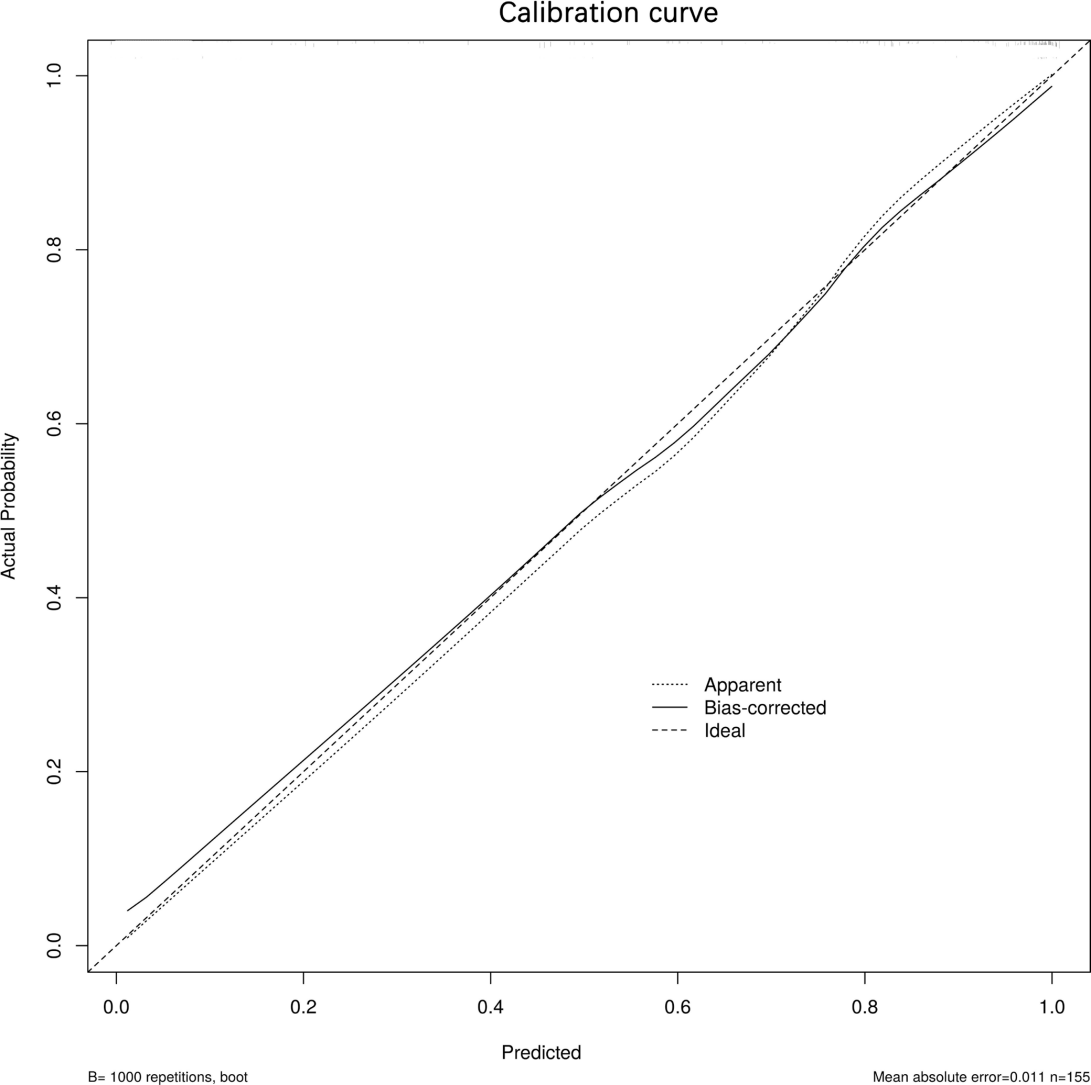
Calibration curve of the nomogram for differentiated thyroid carcinoma (DTC). The x‐axis represents the predicted DTC. The y‐axis represents the actual DTC. The dotted line stands for a perfect prediction using an ideal model. The solid line was drawn to represent the performance of the nomogram, of which the closer fit to the dotted line represents the better prediction of the nomogram.

Utilizing the independent risk factors derived from the multivariate logistic regression analysis—namely nicotinamide, 3‐HAA, 5‐HTP, melatonin, and IPA—a diagnostic nomogram for DTC was developed (Figure 1). The Hosmer–Lemeshow goodness‐of‐fit test did not reveal any statistically significant discrepancies (*p* = 0.589), affirming the model's robust fit. Internal validation of the model's predictive accuracy was conducted through 1000 bootstrap resampling iterations using the original dataset, yielding a calibration curve (Figure 2). The close alignment of the standard curve (dashed line) with the calibration curve (solid line) substantiates the model's predictive accuracy for DTC incidence, underscoring its reliability.

### 3.5 Assessment of the Diagnostic Accuracy of the Five‐Metabolite Panel Model

**FIGURE 3:**
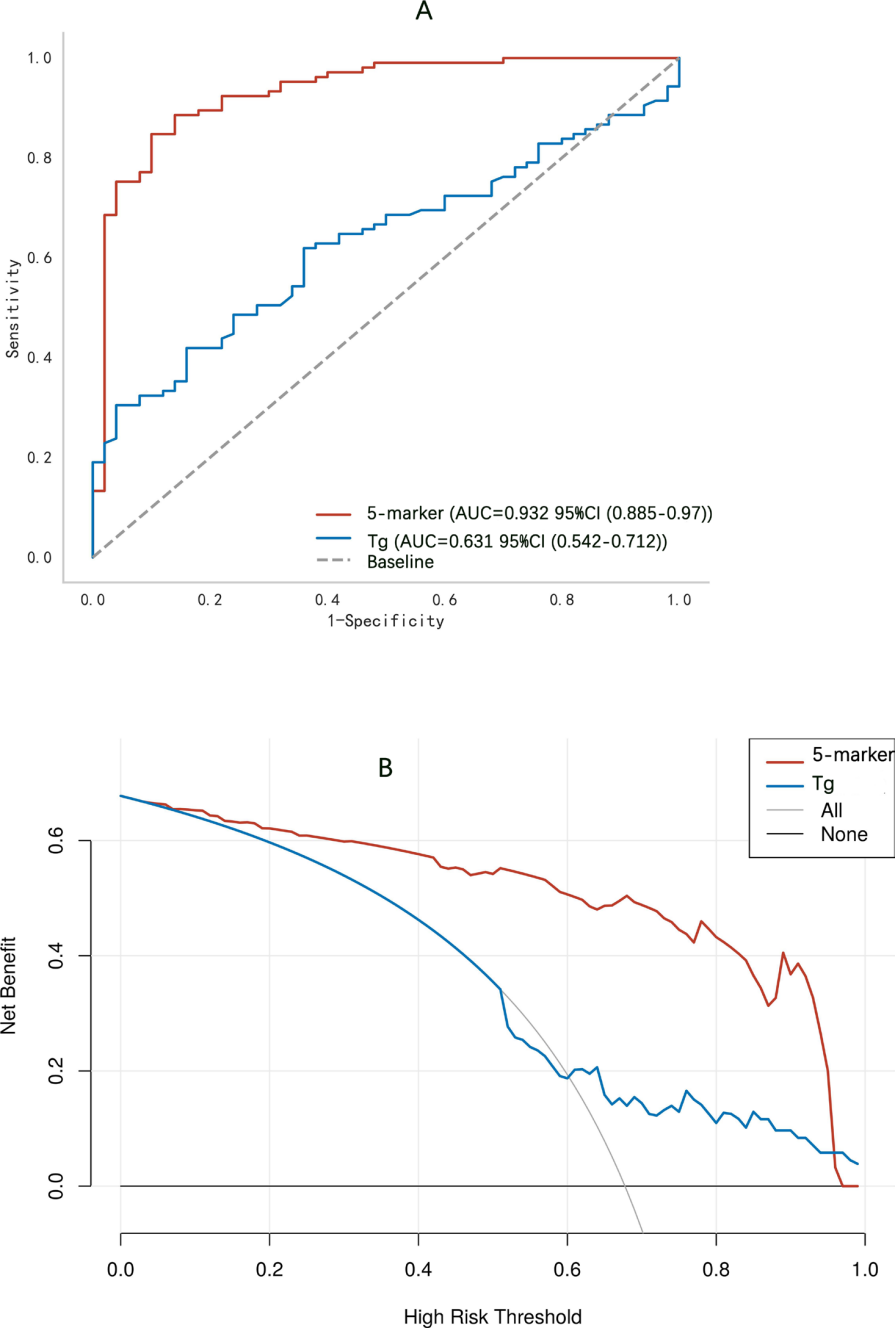
Five‐metabolite panel for early diagnosis of differentiated thyroid carcinoma (DTC). A. ROC analysis of the five‐metabolite panel versus thyroglobulin (Tg) for predicted DTC (*p* < 0.001, Delong test). The values of AUCs and their confidence intervals (CIs) for five‐metabolite panel and Tg are shown, respectively. B. Decision curve analysis for DTC. The black line (none) represents the assumption of net benefit that no patient has DTC. The gray line (All) shows the assumption of net benefit that all patients have DTC. The red and blue line represents the assumption of net benefit for DTC considering five‐metabolite panel (3‐HAA, 5‐HTP, IPA, melatonin, and nicotinamide) and Tg.

The diagnostic efficacy of 11 distinct tryptophan metabolites was initially appraised individually using ROC curves. The corresponding data are presented in Figure S1 and Table S1. The areas under the curves (AUCs) for these metabolites spanned from 0.627 to 0.841. Notably, the AUCs for the five metabolites recognized as DTC‐associated factors—nicotinamide, 3‐HAA, 5‐HTP, melatonin, and IPA—were as follows: 0.760 (95% CI 0.679–0.837), 0.721 (95% CI 0.640–0.798), 0.841 (95% CI 0.767–0.904), 0.785 (95% CI 0.708–0.856), and 0.652 (95% CI 0.555–0.742). Subsequently, the diagnostic potency of the widely utilized serum tumor marker for thyroid cancer, Tg, was evaluated, yielding an AUC of 0.631 (95% CI 0.542–0.712) (Figure 3A). When compared with individual metabolites and Tg, the five‐metabolite panel demonstrated significantly improved diagnostic performance for DTC, with an AUC of 0.932 (95% CI 0.885–0.970), a sensitivity of 0.848, and a specificity of 0.900 (Figure 3A and Table S1). Furthermore, decision curve analysis revealed that the net benefit provided by the five‐metabolite panel substantially exceeded that of Tg, highlighting the superior clinical applicability of this diagnostic approach (Figure 3B).

## 4 Discussion

The thyroid gland plays a fundamental role in human metabolism, regulating energy balance, thermogenesis, and cardiovascular functions through the secretion of thyroid hormones. Even subtle alterations in thyroid function can influence metabolic pathways, including lipid and glucose metabolism, which are critical in both normal physiology and disease states. Recent studies suggest that even in patients without overt thyroid disease, thyroid hormone levels can impact anthropometric measurements and body composition, as well as subclinical cardiovascular function [25, 26]. This highlights the broader metabolic and systemic effects of thyroid function. Thyroid cancer is among the most prevalent malignant tumors of the head and neck region, with its incidence notably on the rise in China in recent years [27, 28]. Despite the generally favorable prognosis for patients with DTC, a subset still faces a significant risk of recurrence and distant metastasis, particularly when diagnosed at advanced stages [29]. The sensitivity and specificity of existing serum markers, such as Tg, are limited, especially in the early diagnostic phase [30]. Tg levels can be affected by factors such as residual thyroid tissue, iodine intake, and autoimmune thyroid conditions, limiting its utility in detecting early‐stage DTC. Additionally, fine‐needle aspiration biopsy (FNAB), though widely used, may yield inconclusive or false‐negative results, particularly in indeterminate nodules. This highlights an urgent need for the development and validation of novel biomarkers and diagnostic models. Our study presents a pioneering systematic analysis of the tryptophan metabolic profile in DTC patients, utilizing LC–MS/MS targeted metabolomics. The study has identified several potential new biomarkers, characterized by significant alterations in serum concentrations. Specifically, the levels of QA, 3‐HAA, 3‐HK, Kyn, Trp, 5‐HTP, melatonin, and IAld were observed to be substantially elevated, while nicotinamide, NAS, and IPA levels were markedly decreased in DTC patients. The abnormal expression of these metabolites may offer novel avenues for the early diagnosis of DTC. Furthermore, multivariate logistic regression analysis identified nicotinamide, 3‐HAA, 5‐HTP, melatonin, and IPA as independent factors influencing the occurrence of DTC. A predictive model incorporating these factors was constructed and demonstrated superior diagnostic performance with an AUC of 0.932, outperforming the conventional tumor marker Tg in clinical decision‐making potential. It is a promising noninvasive tool for DTC detection. By integrating this metabolite panel into routine screening protocols, clinicians may be able to improve risk stratification for patients with thyroid nodules and reduce unnecessary invasive procedures, such as repeated FNAB.

Previous research has begun to establish the diagnostic value of tryptophan metabolites in various cancers. For instance, Onesti et al. observed an increase in tryptophan metabolism among breast cancer patients compared to healthy controls, with significant differences in plasma tryptophan and kynurenine/tryptophan ratios in hormone receptor‐negative patients [31]. Similar findings have been reported in gastric cancer, highlighting the diagnostic potential of the kynurenine/tryptophan ratio [32]. In colorectal cancer, elevated levels of tryptophan and its metabolites have been linked to improved survival rates, suggesting their potential as biomarkers [33]. In line with these studies, our investigation also revealed notable variations in the concentrations of multiple tryptophan metabolites between DTC patients and healthy controls. This discovery underscores the potential clinical utility of tryptophan and its metabolic pathways in the diagnostic and therapeutic realm of DTC, positing these metabolites as prospective biomarkers for the condition.

3‐HAA, an intermediate metabolite of tryptophan critical for the biosynthesis of bioactive molecules, has been identified as an independent influencing factor with significantly elevated levels in DTC patients in our study. Previous research has suggested that 3‐HAA may inhibit ferroptosis in tumor cells, promote tumor growth, and could be a target for cancer therapy [19]. It may also modulate immune responses and promote apoptosis in liver cancer cells, affecting treatment sensitivity [34]. In light of the findings from this study, it is evident that 3‐HAA may exert a significant regulatory influence in DTC, highlighting its potential as a diagnostic biomarker. 5‐HTP and melatonin, also identified as independent influencing factors for DTC diagnosis, are significantly elevated in DTC patients. 5‐HTP, involved in mood regulation and immune response [35], has been associated with hepatocellular carcinoma (HCC), with its serum and tissue levels correlating with patient survival rates [36]. Melatonin, as an important antioxidant and immunomodulator, has received much attention for its role in cancer. Previous studies have found that melatonin exhibits antitumor effects in various cancer types, and its elevation may be related to tumor cell stress response and immune evasion [37]. Its potential as a biomarker for prostate cancer and HCC has been reported [38]. Shang et al. identified galactitol, sucrose, and melatonin as promising candidates for biomarkers in the metabolomic diagnosis of papillary thyroid carcinoma [39]. In line with these findings, the current study supports the emerging notion that 5‐HTP and melatonin could be considered prospective biomarkers for oncological applications.

This study also reports a significant reduction in nicotinamide levels in DTC patients, contrasting with previous findings. Nicotinamide is known for its antitumor activity in certain types of cancer. Yu et al. reported that in the gastric cancer microenvironment, macrophages and fibroblasts remodel CD8+ T cell activity through nicotinamide metabolism [40]. In our investigation, the diminished levels of nicotinamide were identified as an independent influencing factor, potentially reflecting the distinctive metabolic traits of DTC. Given its role as a precursor to NAD+, nicotinamide is integral to energy metabolism and the repair of DNA. The observed reduction may disrupt the metabolic status and genomic integrity of tumor cells, potentially fueling oncogenesis and tumor progression. IPA, a metabolite with antioxidant and anti‐inflammatory effects, has shown significant clinical value in various conditions [41, 42]. The decreased levels of IPA in DTC patients may indicate oxidative stress and inflammation within the tumor microenvironment, presenting a new diagnostic biomarker opportunity.

Multi‐indicator combination models have proven superior to single markers in tumor diagnosis due to their comprehensive biological information and reduced limitations. Gu et al. reported an integrated model achieving high AUCs for early colorectal cancer and advanced adenomas [43]. Wang et al. established a metabolite model from uterine fluid for early ovarian cancer detection with a high AUC [44]. Metabolomics‐based studies have significantly advanced the understanding of thyroid cancer's metabolic characteristics, offering new diagnostic markers. Ryoo et al. identified seven metabolites through metabolomic analysis of fine‐needle aspiration samples from papillary thyroid carcinoma and benign nodules, with AUC values ranging from 0.64 to 0.85 [45]. Chen et al. employed serum and urine metabolomics to screen for six differential metabolites, which were subsequently validated for their diagnostic value for papillary thyroid carcinoma using a logistic regression model. This approach yielded an AUC of 0.943 for female samples and 0.952 for overall samples [46]. Zhang et al. explored amino acid metabolites in saliva, selecting a panel consisting of alanine, valine, proline, and phenylalanine. This panel demonstrated an AUC of 0.936, along with a sensitivity of 91.2% and a specificity of 85.2%, thereby enhancing the precision of early TC diagnosis [47]. Our study, focusing on the tryptophan metabolic profile, constructed a five‐metabolite diagnostic model for DTC, demonstrating superior diagnostic performance with an AUC of 0.932. Decision curve analysis revealed a better net benefit for the model compared to Tg, emphasizing its clinical utility.

In summary, our study demonstrates that tryptophan metabolism is significantly altered in DTC patients and that these metabolic changes can be leveraged for early detection. Given the limitations of existing diagnostic methods, our findings underscore the potential of metabolomics‐based biomarker panels to provide an accurate and noninvasive diagnostic alternative, particularly for early‐stage DTC. However, this study has several limitations. Firstly, it is a single‐center study. Secondly, most subjects were in the early stages of the disease. While this underscores the benefits of early diagnosis, the diagnostic efficacy for mid‐ and late‐stage cases requires further investigation. Lastly, due to the limited number of cases, external cohort validation was not performed. Future research should aim to increase the sample size and conduct multicenter validation to improve external validity.

## 5 Conclusion

This study presents an analysis of the tryptophan metabolic profile in DTC patients, revealing characteristic alterations in tryptophan and its metabolites. A diagnostic model comprising five metabolites—nicotinamide, 3‐HAA, 5‐HTP, melatonin, and IPA—was established, demonstrating enhanced efficacy in early DTC identification and highlighting significant clinical utility.

## Author Contributions

**Qiang Miao:** data curation (equal), writing – original draft (equal). **Xinhua Dai:** methodology (equal). **Xiaojuan Wu:** formal analysis (equal), software (equal). **Li Luo:** data curation (equal), resources (equal). **Junlong Zhang:** project administration (equal), validation (equal). **Han Luo:** conceptualization (equal), writing – review and editing (equal). **Bei Cai:** project administration (equal), writing – review and editing (equal).

## Ethics Statement

The study protocol was approved by the Ethics Committee of the West China Hospital, Sichuan University (No. 2020–823), and adhered to the Helsinki Declaration; all participants provided informed consent.

## Conflicts of Interest

The authors declare no conflicts of interest.

## Supporting information


Data S1.

## Data Availability

Data sharing does not apply to this article, as no new data were created or analyzed in this study.
