# Supplementary material for: Analysis of Tryptophan Metabolic Profile Characteristics and Clinical Value in Differentiated Thyroid Cancer Patients
Source: Cancer Med. 2025 Mar 25;14(6):e70808. doi: 10.1002/cam4.70808 (PMC11933853; doi:10.1002/cam4.70808)
Supplement: Supplementary file 1 — Data S1. [file CAM4-14-e70808-s001.docx]

**Analysis of tryptophan metabolic profile characteristics and clinical value in patients with differentiated thyroid cancer**

| **Table S1 Area under the ROC curve** | | | | | |
| --- | --- | --- | --- | --- | --- |
| **Features** | **AUC** | **Sensitivity** | **Specificity** | **Y-index** | **Cut-off** |
| Nicotinamide (nmol/L) | 0.760 | 0.86 | 0.629 | 0.489 | 288.8 |
| QA (nmol/L) | 0.627 | 0.314 | 0.900 | 0.214 | 288.9 |
| 3-HAA (nmol/L) | 0.721 | 0.600 | 0.800 | 0.400 | 21.31 |
| 3-HK (nmol/L) | 0.718 | 0.524 | 0.820 | 0.344 | 31.41 |
| Kyn (nmol/L) | 0.673 | 0.724 | 0.540 | 0.264 | 1706 |
| Trp (μmol/L) | 0.796 | 0.771 | 0.720 | 0.491 | 54.58 |
| 5-HTP (nmol/L) | 0.841 | 0.733 | 0.840 | 0.573 | 2.807 |
| NAS (nmol/L) | 0.723 | 0.820 | 0.571 | 0.391 | 0.037 |
| Melatonin (nmol/L) | 0.785 | 0.610 | 0.920 | 0.530 | 0.071 |
| IPA (nmol/L) | 0.652 | 0.500 | 0.790 | 0.290 | 1189 |
| IAld (nmol/L) | 0.777 | 0.924 | 0.560 | 0.484 | 119.5 |
| 5-metabolite panel | 0.932 | 0.848 | 0.900 | 0.748 | / |
| Abbreviation: ROC, Receiver operator characteristic curve; AUC, Area under the curve; QA, Quinolinic Acid; 3-HAA, 3-Hydroxyanthranilic Acid; 3-HK, 3-Hydroxykynurenine; Kyn, Kynurenine; Trp, Tryptophan; 5-HTP, 5-Hydroxytryptophan; NAS, N-Acetylserotonin; IPA, Indole-3-Propionic Acid; IAld, Indole-3-Aldehyde; 5-metabolite panel, Includes 5 tryptophan metabolites (3-HAA, 5-HTP, IPA, melatonin and nicotinamide). | | | | | |

**Figure S1.** ROC analysis of the 11 individual metabolites (Nicotinamide, QA, 3-HAA, 3-HK, Kyn, Trp, 5-HTP, NAS, Melatonin, IPA, IAld). The value of AUC for each metabolite is shown.

**
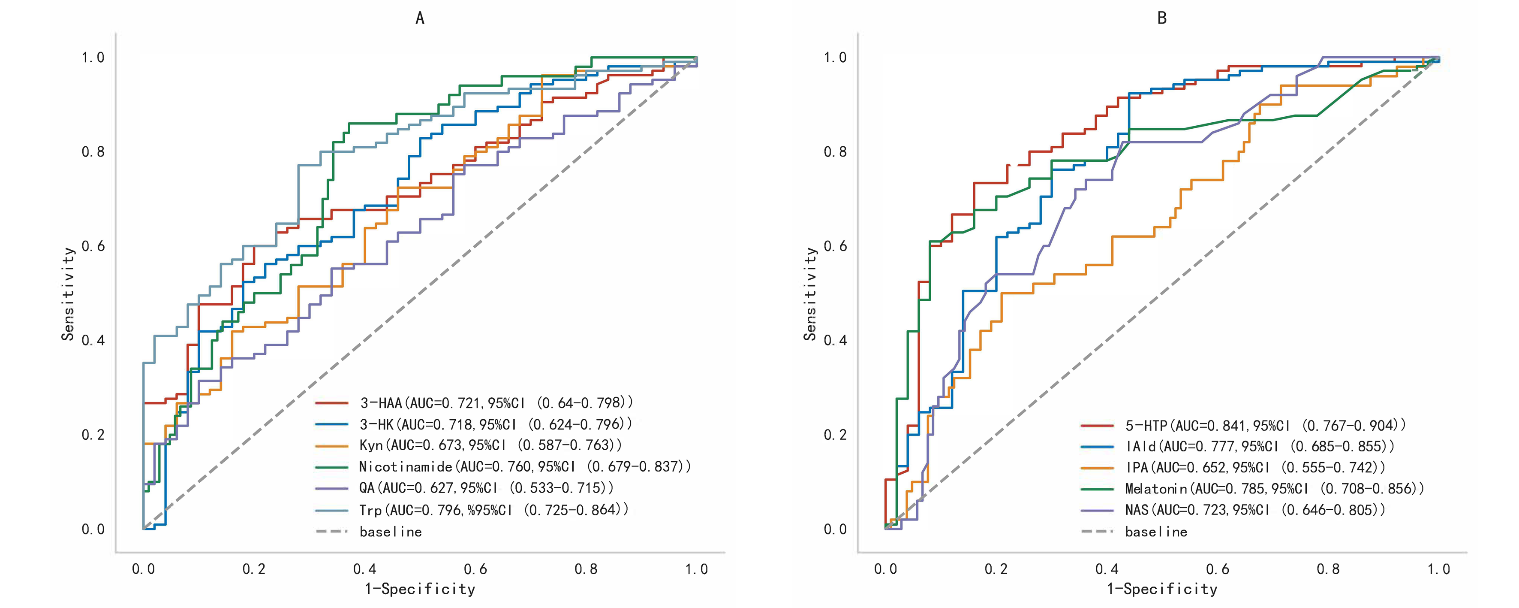
**
